# Urban development trend analysis and spatial simulation based on time series remote sensing data: A case study of Jinan, China

**DOI:** 10.1371/journal.pone.0257776

**Published:** 2021-10-07

**Authors:** Yanghua Zhang, Liang Zhao, Hu Zhao, Xiaofeng Gao

**Affiliations:** 1 School of Architecture and Urban Planning, Shandong Jianzhu University, Jinan, China; 2 School of Civil Engineering, Qingdao University of Technology, Qingdao, China; Northeastern University (Shenyang China), CHINA

## Abstract

Uncontrolled urban growth detracts from healthy urban development. Understanding urban development trends and predicting future urban spatial states is of great practical significance. In order to comprehensively analyze urbanization and its effect on vegetation cover, we extracted urban development trends from time series DMSP/OLS NTL and NDVI data from 2000 to 2015, using a linear model fitting method. Six urban development trend types were identified by clustering the linear model parameters. The identified trend types were found to accurately reflect the on-ground conditions and changes in the Jinan area. For example, a high-density, stable urban type was found in the city center while a stable dense vegetation type was found in the mountains to the south. The SLEUTH model was used for urban growth simulation under three scenarios built on the urban development analysis results. The simulation results project a gentle urban growth trend from 2015 to 2030, demonstrating the prospects for urban growth from the perspective of environmental protection and conservative urban development.

## Introduction

Global urbanization has accelerated in recent decades. The average urbanization level is expected to reach 86% in developed countries and 64% in developing countries by 2050 [1]. Although urbanization is commensurate with socioeconomic development, it also causes problems such as cropland loss, deforestation, regional climate and environmental impacts [2–5]. In order to achieve sustainable development and support rational urban planning and management, it is of great practical significance to understand urban development trends and predict future urban spatial configurations.

In recent decades, remote sensing data have been widely used in urbanization process studies because, in comparison with census data, they can provide timely and spatially explicit information [6, 7]. Frequently used remote sensing data include multispectral reflectance [8, 9], normalized difference vegetation index (NDVI) [10, 11], nighttime lights data (NTL) [7, 12], the biophysical composition index (BCI) [13] and other indicators that reflect urban land types [14]. Most of these data have been used for detection of urban land use change. Two types of detection methods have been developed, namely temporal trajectory analysis and post-classification [15]. The former typically builds models using time series remote sensing products to analyze change trends in urban land. These land changes include inter-class changes, between different urban land types, such as between farmland or forest and built-up land [16, 17], and intra-class changes, such as changes in built land density or in vegetation coverage [18, 19]. The post-classification method first classifies land use/cover types at each time, and then analyzes land changes by comparing the time series classifications [20, 21]. Nowadays, with developments in remote sensing and deepening concern for sustainable development, combinatorial analysis of urbanization processes and ecological trends, using time series remote sensing data, has become very important.

As described above, change trends in urban land have been previously extracted from time series data. Since human beings cannot change their past, the main objective of these studies has been to assist in future rational exploitation and utilization of urban land. To this end, many scholars have focused on studies simulating future urban land distributions [2, 22, 23]. Various types of models and methods have been proposed and used in urban land simulations [22, 24]. Among these models, the majority are based on cellular automata (CA) because they have been shown to be effective for representing and simulating the complexity of the dynamic processes involved in urban growth and land use change [25–27]. In recent decades, CA modeling has been advanced by significant technological innovations, and many CA-based urban models have been developed, including UrbanSim [28], ANN-CA [29], RF-CA [30], SLEUTH [31], CLUE-S [32], and FLUS [23]. These models are commonly applied under various urban development scenarios, and allow comparison of simulation results under the different scenarios [23, 24]. Then, urban development suggestions have been proposed based these comparisons. The existing research has shown these models to be effective in this regard.

Since the reform and opening up, China has experienced rapid urbanization. In particular, many eastern coastal cities have developed into metropolises with populations of more than ten million. Jinan, the capital of Shandong Province, which is the third largest economic aggregation in China, also developed rapidly. From 2000 to 2016, driven by the real estate economy, the urban area of Jinan expanded by 37% [33]. However, this mode of urban development is unhealthy and unsustainable, and leads to cropland loss and environmental disruption. To remedy this development situation, the government has generally attached great importance to environmental protection. As a representative eastern Chinese city, Jinan has been selected as the study area in many urban expansion and urban simulation studies [33–35]. With the deepening concern for the environment, combinatorial analysis of urbanization processes and ecological trends is now needed to support sustainable future development of Jinan.

In previous studies, time series NDVI and NTL data have been used for urban land use extraction [36, 37] and urban land dynamic analysis [38–40]. Correlation analysis is usually used to explore the influence of urbanization on the eco-environment [38, 39]. However, while correlation analysis provides the degree of correlation between urbanization and ecological degradation, it is difficult to determine urban development trend types, and the pace of change and spatial distribution patterns of these types. Therefore, in this study, we used time series remote sensing data to analyze Jinan’s development trends in urbanization and ecology and summarized the development types and simulated future urban expansion under a number of scenarios based on the combinatorial analysis.

## Study area and data

### Study area

**Fig 1:**
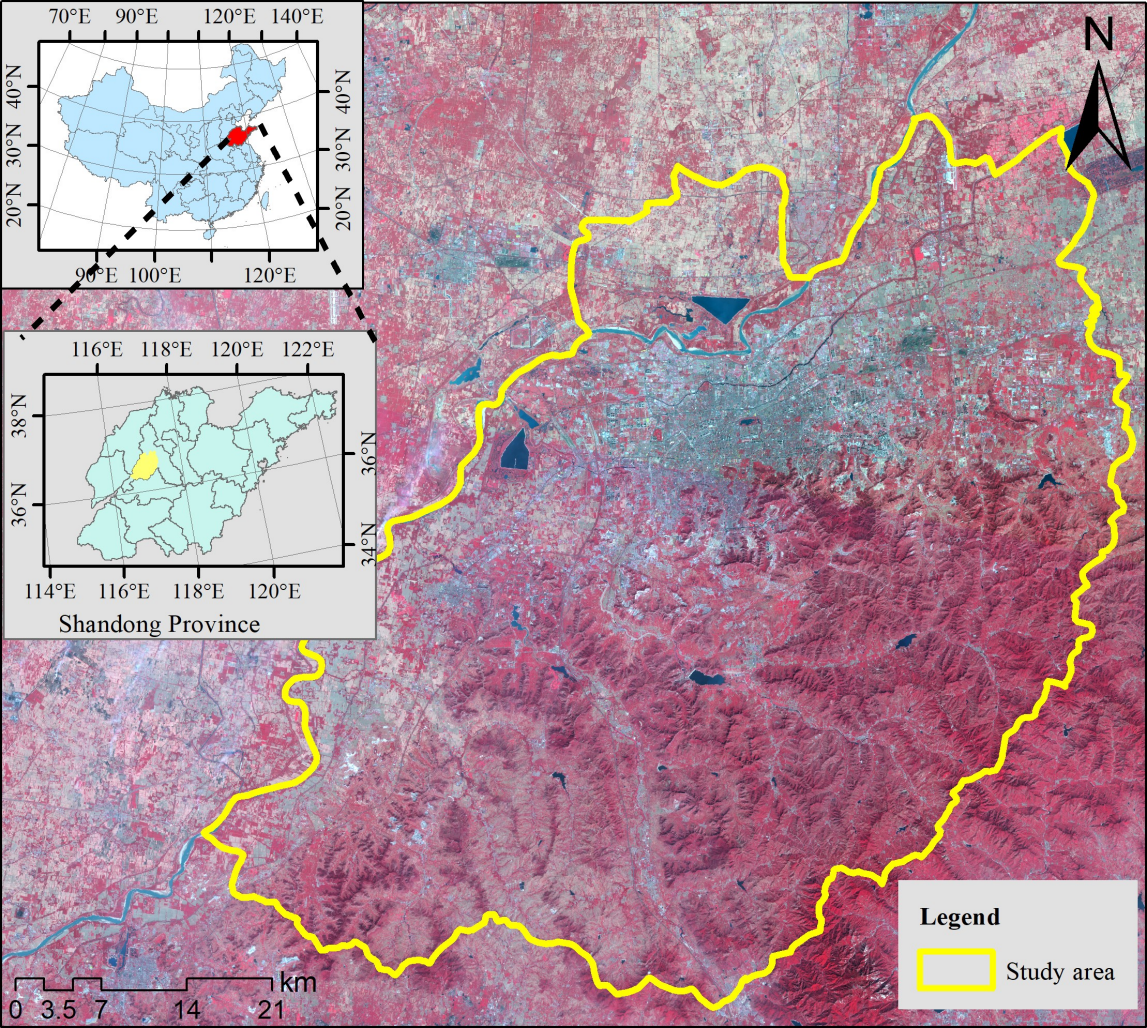
Location of the study area, mainly included urban area and suburb areas.

Jinan, as the cultural, commercial, financial, and political center of Shandong Province, China, has experienced rapid development. From 2006 to 2018, the built-up land area of Jinan grew from 305 km^2^ to 524 km^2^. The main urban areas and some fast-developing suburbs were selected for this study. The geographical position is between 36°14´ N–36°54´ N and 116°30´ E–117°22´ E. As shown in **Fig 1**, the study area included Shizhong District, Lixia District, Licheng District, Tianqiao District, Huaiyin District, and Changqing District. In recent decades, the urban expansion of Jinan has occurred mainly in these areas.

### Data and pre-processing

The data primarily used in this study were time series of Defense Meteorological Satellite Program/Operational Linescan System (DMSP/OLS) nighttime stable light data (NTL) data from 2000 to 2013, normalized difference vegetation index (NDVI) data from 2000 to 2015, urban land cover data from 2000 to 2015, a water mask, traffic network vector data, and a digital elevation model (DEM).

The DMSP/OLS NTL time series data were downloaded from the NOAA National Centers for Environmental Information (NCEI) website (https://www.ngdc.noaa.gov/ngdc.html). The digital numbers (DN) in the NTL data reflect the urbanization level, with high values in the center of the city, low values in peripheral regions, and values close to zero in uninhabited regions [41]. NTL is widely used in dynamic monitoring of urban expansion, economic development, and population evolution [6, 41, 42]. Thus, we selected time series NTL data for urbanization analysis of the study area. The NTL data have a spatial resolution of 30 arc-seconds and a DN range of 0–63. The calibration method developed by [43] was used to improve the inter-annual comparability of the time series NTL data. The NTL data from the middle year, 2007, were selected as the reference image, and Mauritius, Puerto Rico, and Okinawa were selected as invariant regions [12]. To reduce the effect of NTL saturation, the Vegetation Adjusted NTL Urban Index (VANUI) [44] was used in calibrating NTL values based on time series NDVI images. Lastly, the NLT time series DN values were normalized to the range of 0–1.

The time series NDVI data collected by SPOT/VEGETATION were downloaded from the Resource and Environment Science and Data Center of China (http://www.resdc.cn/). NDVI is an effective vegetation indicator whose value ranges from –1 to 1. Denser vegetation has NDVI value closer to 1. It is widely used in long-term change detection of forests, grasslands, farmlands, and other vegetated areas [45–47]. Thus, time series NDVI was therefore chosen to identify change trends in the ecology of the study area. Composite NDVI, from August 2000 to 2015, was selected because August is a time of flourishing vegetation, and therefore shows the greatest difference between land types. For comparability with the NTL data, the time series NDVI was also normalized to the range of 0–1.

Urban land cover data were downloaded from an open source repository [48]. These data were derived from Landsat images with a spatial resolution of 30 m and four years were selected for urban simulation, namely 2000, 2005, 2010, and 2015. The digital elevation model (DEM) was downloaded from the Geospatial Data Cloud website (http://www.gscloud.cn/) and used to derive slope and hillshade data. Finally, all raster and vector data were clipped to the same extent as the study area, and the projection coordinate system was uniformly set to WGS_1984_UTM_Zone_50N.

## Methodology

**Fig 2:**
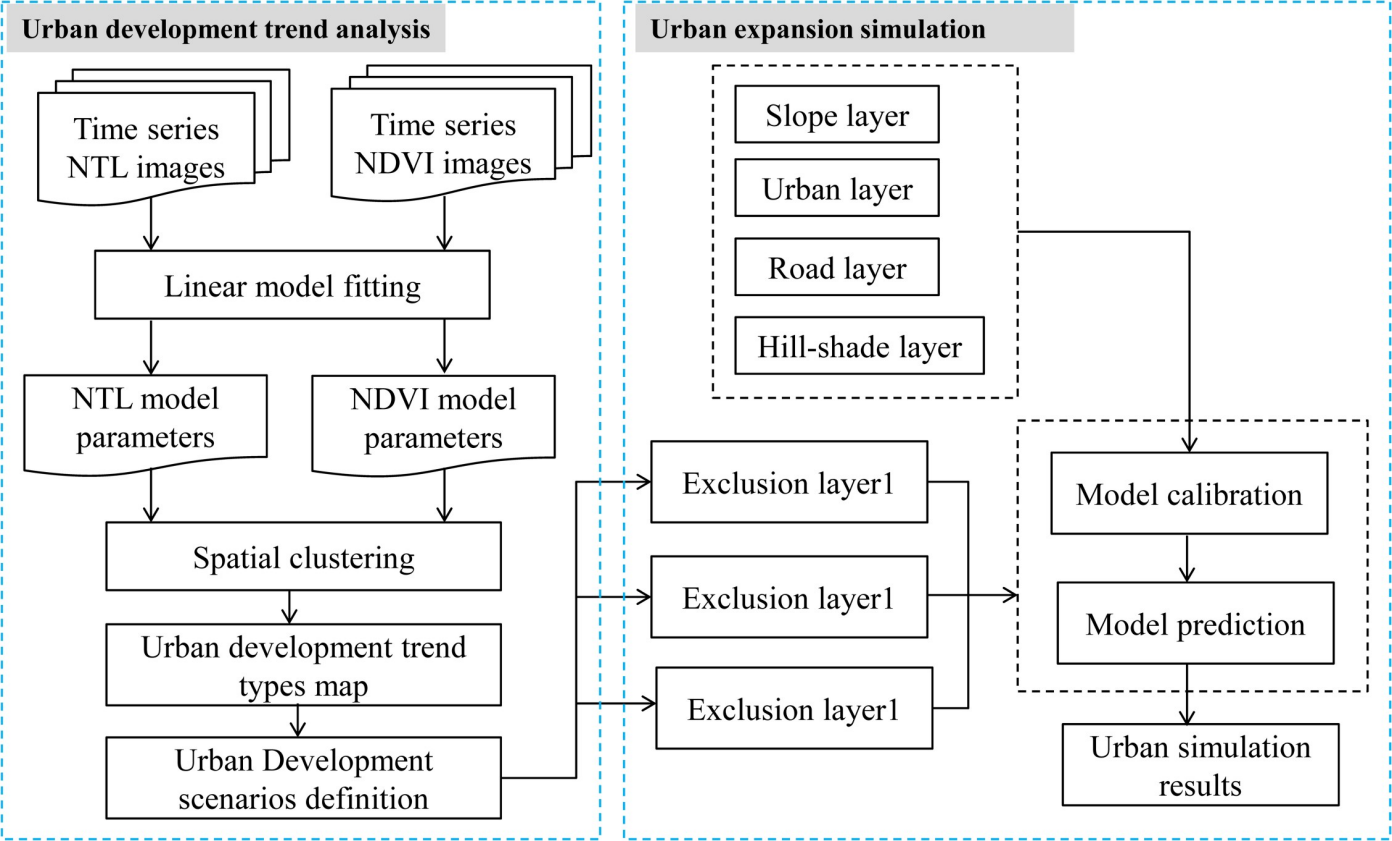
The flowchart of this study.

The methodology of this work included two main parts: (1) urban development trend analysis and (2) urban simulation. The former synthetically analyzed the urbanization process and ecological change trend from 2000 to 2015 using time series NTL and NDVI data, and then identified a set of urban development spatial area types based on a spatial clustering procedure. The latter simulated the urban area in 2030 under different scenarios based on the prior analysis. The detailed methodology is shown in **Fig 2**.

### Urban development trend analysis

Change trend analysis of time series NTL and NDVI data is usually a model-fitting process using linear, non-linear, or harmonic models [12, 18, 49, 50]. Linear models are commonly used in general change trend analysis and do not consider local changes. Nonlinear models are often used in detailed change trend analysis and include local changes. Harmonic models are typically used in seasonal change analysis. Because this study aimed to define general change trends in NTL and NDVI, a linear model was applied to the time series.

**Fig 3:**
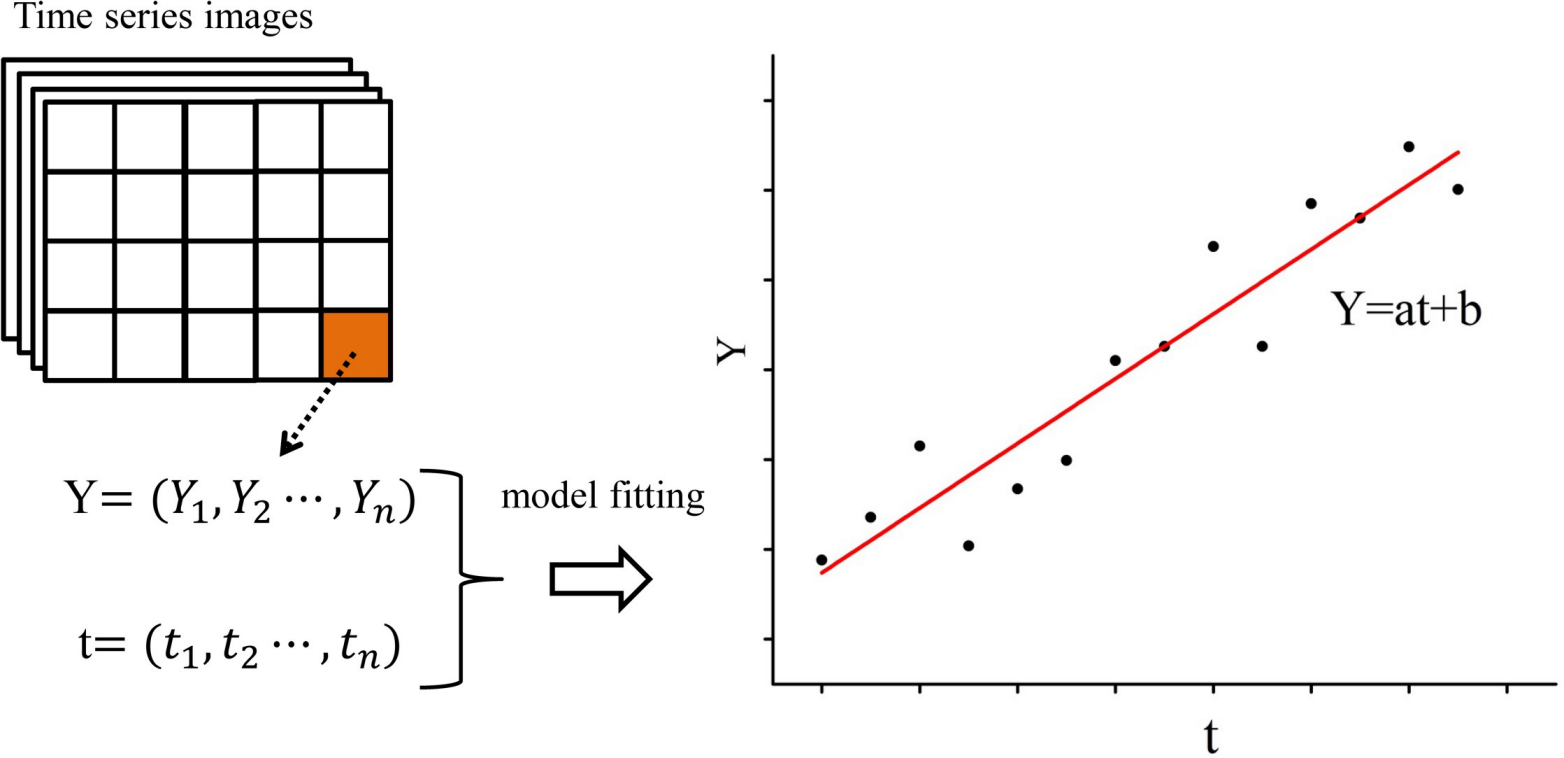
Illustration of linear model fitting of time series values.

The linear model was built using least-squares first-order regression of the time series values of each image pixel. Thus, every pixel of the image had an individual model, as shown in **Fig 3** (*Y = at+b*). *Y* denotes the time series values of normalized NTL or NDVI, *t* denotes the time points, *a* denotes the slope value of the linear model, and *b* denotes the intercept value of the linear model. For example, for the *ith* pixel of the NDVI images, the model is *Y*_(*NDVI*,*i*)_ = a_(*NDVI*,*i*)_t + b_(*NDVI*,*i*)_, and for the *ith* pixel of the NTL images, the model is *Y*_(*NTL*,*i*)_ = a_(*NTL*,*i*)_t + b_(*NTL*,*i*)_. The goodness of fit of the models was evaluated using the root mean squared error (RMSE) indicator, which is defined as the square root of the mean of the squared differences between corresponding elements of the model values and actual values [51]. The smaller the RMSE value, the better the goodness of fit.

As shown in **Fig 3**, in a model, the value of parameter *a* reflects the change trend and the speed of change, and the parameter *b* reflects the size of the initial values of the time series. If *a* is a negative value, the change trend is declining. If *a* is a positive value, the change trend is increasing. If the absolute value of *a* is large, the speed of change is fast. If the absolute value of *a* is small, the change speed is slow. Thus, it can be seen that the change trend of time series data can be summarized from the two parameters, namely *a* and *b*. To synthetically analyze the evolution trend of NTL and NDVI, vectors were constructed based on NTL and NDVI model parameters, specifically *V* = 〈*a*_*NTL*_, *b*_*NTL*_, *a*_*NDVI*_, *b*_*NDVI*_〉. Then, the vectors of all pixels were clustered, based on their similarity, using the k-means clustering algorithm. First, the number of clusters (k) and a set of n data points were selected as the original cluster centers. Then, the sum of the distances between each point and its closest center (d_sum_) was calculated iteratively. The cluster centers were iteratively optimized to created optimal clusters that minimized the value of d_sum_. Although it is one of the earliest clustering algorithms, it is simple and effective and still popular for a variety of applications [52, 53].

According to the rapid urban expansion state in China in recent decades, we applied the general assumption that the degeneration of urban land is not likely in this study [16]. Thus we defined the change trend of urban land as three types, namely, urban expansion type, high density urban stable type and low density urban stable type, and defined the change trend of vegetation as four types, namely, the type of vegetation coverage increase, the type of vegetation degeneration, high density vegetation stable type and low density vegetation stable type. Based on these definitions of urban and vegetation change trend type, there are totally twelve urban development trend types under the comprehensive consideration of urban land and vegetation. Therefore we set the initial cluster number as twelve.

Considering that there may be some types are not exist in the study area. After the clustering procedure, each cluster’s urban development trend type was determined using sample data analysis. The detailed scheme was as follows. First, some sample pixels within each cluster region were randomly selected. Then, the linear model parameters of the sample pixels were extracted by least-squares first-order regression. Lastly, the development trend of each cluster was defined based on the mean values of the linear model parameters. For example, for one cluster, if the mean value of *a*_*NTL*_ is a large positive value, *b*_*NTL*_ is a small value, *a*_*NDVI*_ is a large negative value, and *b*_*NDVI*_ is a large value, (i.e., NTL increased rapidly while NDVI decreased rapidly) this represents an urban development type in which urban areas have expanded rapidly with a large loss of vegetation.

### Urban simulation

The SLEUTH (Slope, Land use, Exclusion, Urban, Transportation, and Hill-shade) model was selected to simulate urban expansion. The SLEUTH model is a well-known CA-based urban growth model that can predict future urban development areas under different urban growth scenarios. The model uses simple simulation rules and has been refined many times [54]. Its required spatial layers include roads, terrain, existing urban/non-urban areas, land use, and exclusion areas. By changing the exclusion layers, the SLEUTH model can simulate different urban growth scenarios, and many studies have incorporated planning policies and environmental quality constraints into the model using this method. The SLEUTH model has been widely applied and has shown robust capability to simulate the emergence of complex urban patterns [2, 55, 56]. This study only used the SLEUTH-UGM model, thus the land use layer was not included in the input data. Therefore, there are five input layers have been used: slope, exclusions, urban areas, transport, and hillshade. Changing the exclusion layer allows comparison of different scenarios [2, 27]. This process was used in this study for scenario setting and model calibration.

#### Scenario setting

Urban expansion scenarios were set by adjusting the grid cell values in the exclusion layers. The cell values ranged from 0–100, with higher values indicating higher probability of grid cells not being urbanized [24]. For example, water bodies are typically not suitable for urban construction and water cells were given an exclusion value of 100. In an environmental protection scenario, forests and wetlands have a value exclusion probability close to 100. This process also allowed us to define urban expansion scenarios based on results of the urban development trend analysis. For example, for a region with rapid urban expansion at but major vegetation loss, the exclusion layer’s value might be set as close to 100, in order to protect the remaining ecology. A region of rapid urban expansion at little vegetation cost has seen urban expansion with little influence on ecology, meaning it has space for further urban expansion. Thus, the exclusion layer’s values in this region would be set small. In all we applied three scenarios. The first reflects historical growth with high exclusion probability only for water bodies. The other two scenarios were based on outcome of the urban development trend analysis.

#### Model calibration

The SLEUTH model uses four rules to simulate urban growth: spontaneous growth, new spreading center growth, edge growth, and road-influenced growth. To control these four rules, five coefficients, namely dispersion, breed, spread, road gravity, and slope, need to be chosen [57]. To obtain the optimal coefficient values, model calibration is essential. This involves comparison of the simulated and actual urban growth patterns and is a multi-stage, automated, and sequential process using a forced Monte Carlo iterative method and divided into three phases: coarse calibration, fine calibration, and final calibration [24]. The three calibration phases sequentially narrow the ranges of the coefficient values using a predetermined iteration interval and step size. Each coefficient combination is applied in simulating urban growth patterns based on the input data. The degree of fit, under each coefficient combination, between the simulated urban growth and actual urban growth is assessed using indexes based on SLEUTH options such as Compare, Population, Edges, Clusters, Lee-Salle, Slope, X-mean, and Y-mean [54]. Among these indexes, Lee-Salle is widely used [58, 59]. It is a shape index, which quantifies the spatial fit between the model’s growth and the known urban extent. The higher the Lee-Salle value, the better the simulation effect. Therefore, this study selected the Lee-Salle index to assess the performance of each coefficient combination.

## Results

### Urban development trend analysis results

**Fig 4:**
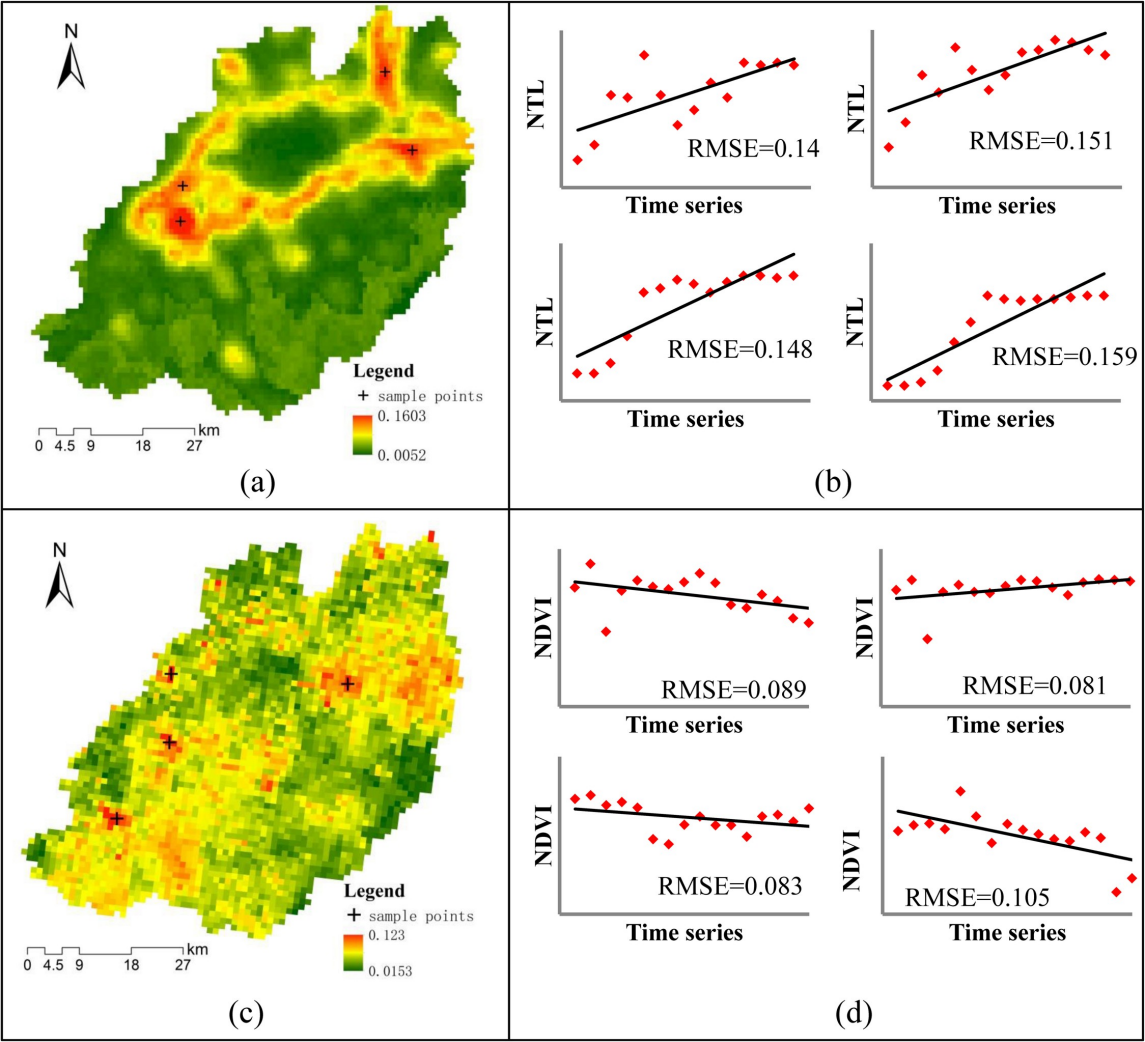
The linear fitting goodness evaluations of NTL and NDVI time series. (a) the RMSE map of the NTL time series; (b) examples of NTL linear fitting at sample points with high RMSE values; (c) the RMSE map of NDVI time series; (d) examples of NDVI linear fitting of sample points with high RMSE values.

The results of the linear fitting goodness evaluation are shown in Fig 4. The RMSE values of the NTL time series ranged from 0.0052 to 0.1603 (**Fig 4A**), and the RMSE values of the NDVI time series ranged from 0.0153 to 0.123 (**Fig 4C**). To further verify the goodness of linear fitting, four sample points in the NTL and NDVI high RMSE areas of were separately extracted, and their linear models are shown in **Fig 4B** and **4D**. From these sample point linear models, we can see that although their RMSE values are higher than those of other areas, they reveal the overall change trend well. Therefore, the linear models were judged as effective for development trend identification, and the resulting model parameters were deemed suitable for the spatial clustering progress.

**Fig 5:**
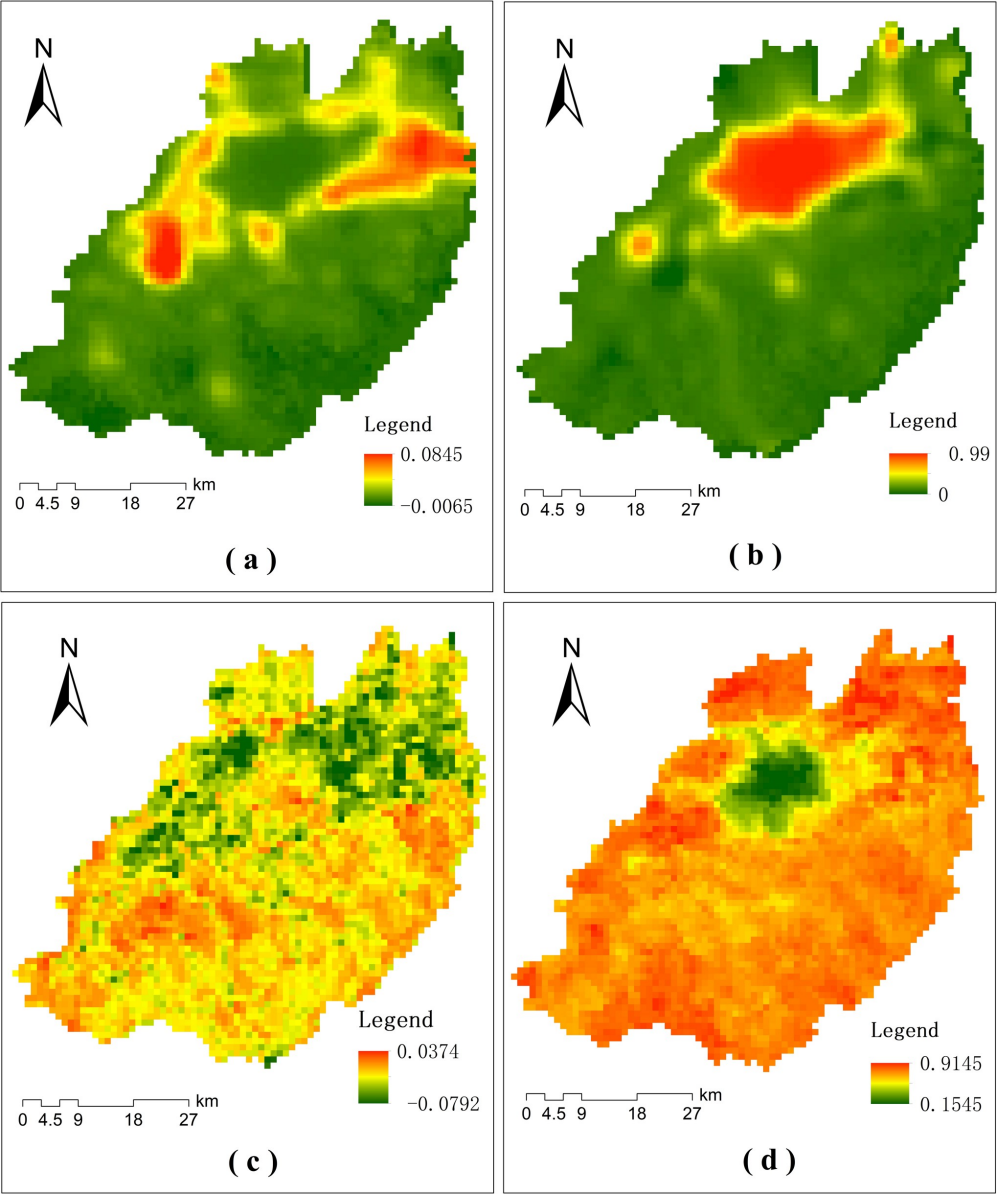
The spatial distributions of the linear model parameters. (a) map of *a*_*NTL*_ values; (b) map of *b*_*NTL*_ values; (c) map of *a*_*NDVI*_ values; (d) map of *b*_*NDVI*_ values.

Maps showing the spatial distribution of the linear model parameters of the NTL and NDVI time series are in **Fig 5**. Low values of *a*_*NTL*_ were mainly in the south of the study area and in the center of the city (**Fig 5A**). This indicates that these areas have experienced slow urbanization. High values of *a*_*NTL*_ were mainly around the central area of the city and to the east and southwest of the central city. Rapid urbanization was mostly in these two directions. High values of *b*_*NTL*_ were mainly in the center of the city and the administrative center of the suburban district (**Fig 5B**) indicating that these areas were mostly urbanized before 2000 and have experienced slow urbanization since then.

Low values of *a*_*NDVI*_ had a similar distribution pattern to high *a*_*NTL*_ values (**Fig 5C**), and were mainly in the eastern and southwestern parts of the city. Urbanization of these areas has clearly influenced vegetation cover. High values of *a*_*NDVI*_ were mainly in the south of the study area, areas of mostly forest and grassland, indicating that the south of the study area has maintained good vegetation coverage and ecological conditions. Low values of *b*_*NDVI*_ were mainly distributed in the center of the city, and high values were mostly in other areas (**Fig 5D**) reflecting good vegetation coverage of most of the study area in 2000.

**Fig 6:**
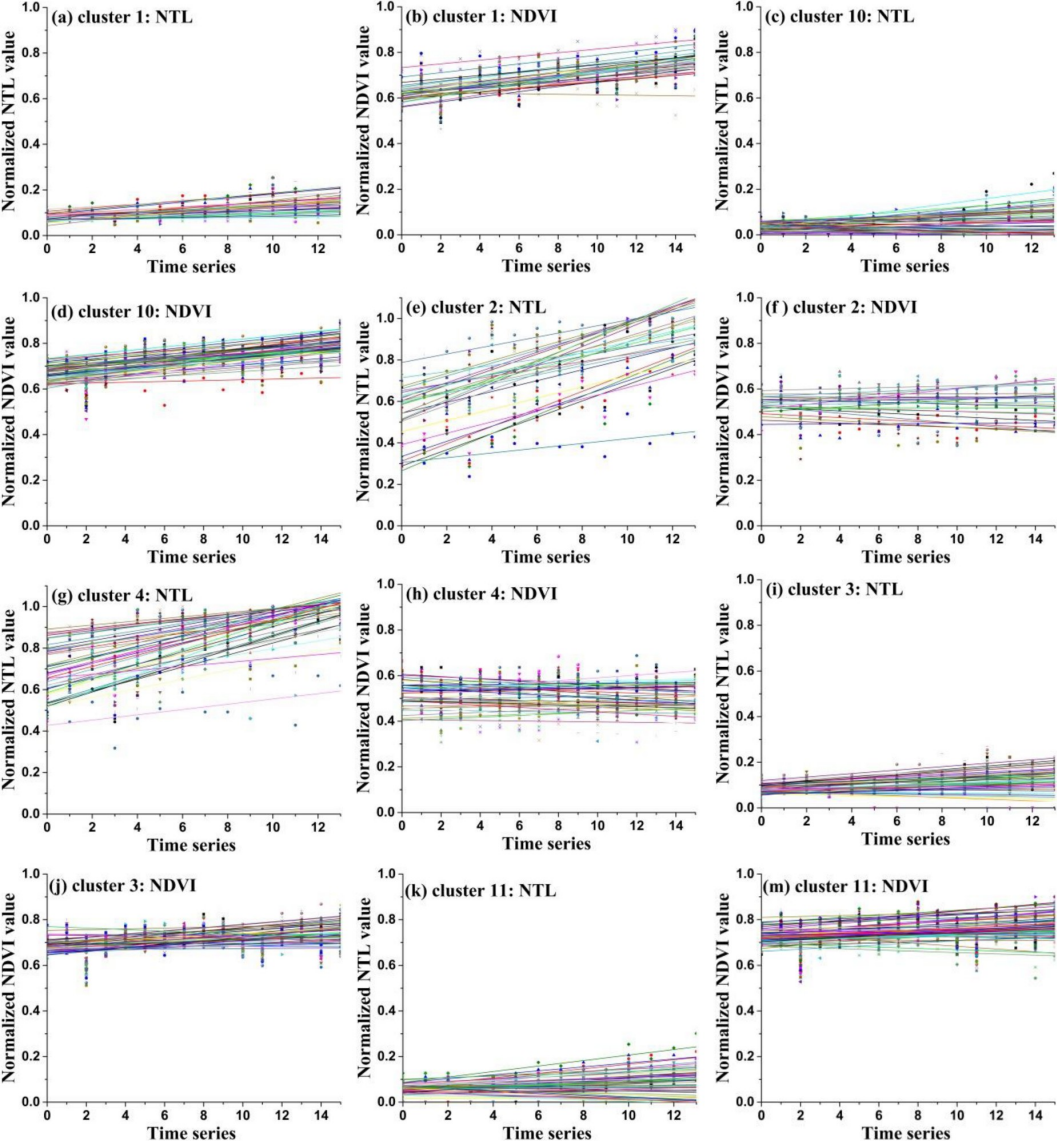
The linear model lines of the samples of cluster 1, cluster 2, cluster 3, cluster 4, cluster 10 and class 11.

**Fig 7:**
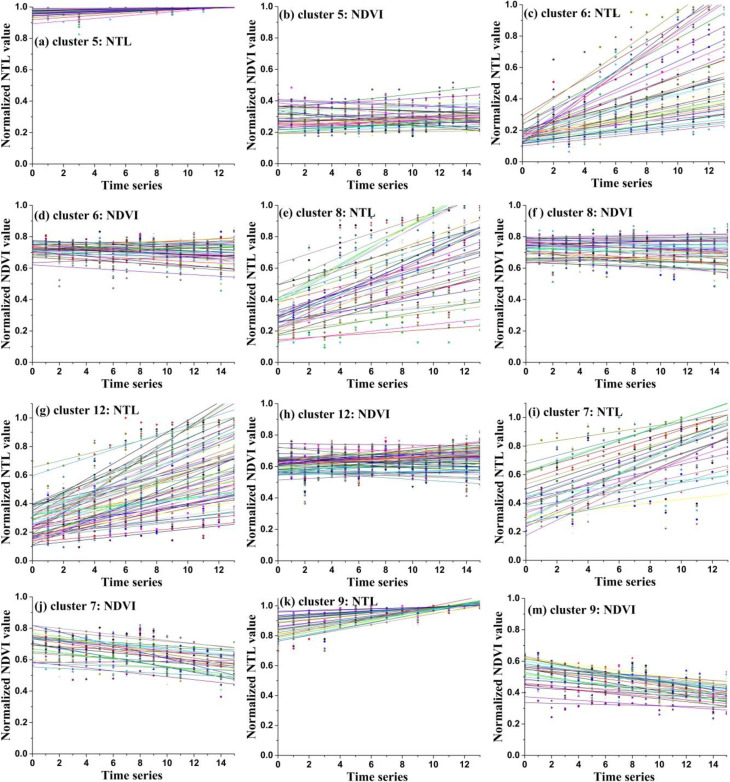
The linear model lines of the samples of cluster 5, cluster 6, cluster 7, cluster 8, cluster 9 and class 12.

As mentioned above, we used time series normalized NTL images from 2000 to 2013 and NDVI images from 2000 to 2015 to build linear models. Four linear model parameters were extracted for each pixel, namely *a*_*NTL*_, *b*_*NTL*_, *a*_*NDVI*_ and *b*_*NDVI*_. Then, the linear model parameters of each pixel were used for spatial clustering. As stated above, the initial cluster number is twelve. The mean values of the four linear model parameters in each cluster region were extracted and are shown in Table 1. And the linear models of each cluster’s samples are shown in **Figs 6** and **7**.

**Table 1:** Results of clustering the linear model parameters and the urban development trends of each cluster.

Cluster number	Mean parameter value of each cluster				Development trend types
	*a*_*NTL*_	*b*_*NTL*_	*a*_*NDVI*_	*b*_*NDVI*_	
1	0.0056	0.0746	0.0172	0.6108	**Type 1:** Low density stable urban and vegetation increase type (LDSU-VI)
2	0.0329	0.5157	0.0045	0.5212	**Type 2:** Urban expansion and Low density stable vegetation type (UE-LDSV)
3	0.0068	0.0851	0.0058	0.6859	**Type 3:** Low density stable urban and High density stable vegetation type (LDSU-HDSV)
4	0.0239	0.6625	-0.0068	0.5131	**Type 2:** Urban expansion and Low density stable vegetation type (UE-LDSV)
5	0.0028	0.96	0.0049	0.2858	**Type 4:** High density stable urban and low density stable vegetation type (HDSU-LDSV)
6	0.0288	0.1522	-0.0049	0.7182	**Type 5:** Urban expansion and high density stable vegetation type (UE-HDSV)
7	0.0322	0.4511	-0.0117	0.6915	**Type 6:** Urban expansion and vegetation degeneration type (UE-VD)
8	0.033	0.2896	-0.0041	0.728	**Type 5:** Urban expansion and high density stable vegetation type (UE-HDSV)
9	0.0106	0.8754	-0.0129	0.5248	**Type 6: U**rban expansion and vegetation degeneration type (UE-VD)
10	0.0076	0.0331	0.0123	0.6626	**Type 1:** Low density stable urban and vegetation increase type (LDSU-VI)
11	0.007	0.0658	0.0033	0.7237	**Type 3:** Low density stable urban and High density stable vegetation type (LDSU-HDSV)
12	0.0268	0.2384	0.007	0.6228	**Type 5:** Urban expansion and high density stable vegetation type (UE-HDSV)

For cluster 1 and cluster 10, the slope values of the normalized NTL lines were small and indicated a stable trend (**Fig 6A** and **6C**). The mean slope values of the normalized NDVI lines were all larger than 0.01 and indicated a increase trend (**Fig 6B** and **6D**). Thus, the development trend type of cluster 1 and cluster 10 was defined appropriately as low density stable urban and vegetation increase type (LDSU-VI). For cluster 2 and cluster 4, the normalized NTL lines exhibited an obvious growth trend (**Fig 6E** and **6G**). The normalized NDVI lines all showed stable trends at roughly median values reflecting the low-density vegetation cover evident in the original NDVI values (**Fig 6F** and **6H**). Thus, the development trend type of cluster 2 and cluster 4 was defined appropriately as the urban expansion and low density stable vegetation type (UE-LDSV). For cluster 3 and cluster 11, the normalized NTL lines presented a stable trend and maintained low values (**Fig 6I** and **6K**), and the normalized NDVI lines also presented a stable trend and maintained high values (**Fig 6J** and **6M**). Thus, the development trend type of cluster 3 and cluster 11 was defined appropriately as low density stable urban and high density stable vegetation type (LDSU-HDSV).

For cluster 5, the normalized NTL lines had a stable trend and maintained high values (Fig 7A). The normalized NDVI lines also had a stable trend and maintained low values (Fig 7B). Thus, the development trend type of cluster 5 was defined appropriately as high density stable urban and low density stable vegetation type (HDSU-LDSV). For cluster 6, cluster 8 and cluster 12, the normalized NTL lines showed an obvious growth trend (**Fig 7C, 7E and 7G**) while the normalized NDVI lines presented a stable trend and maintained high values (**Fig 7D, 7F and 7H**). Thus, the development trend type of cluster 6, cluster 8 and cluster 12 was defined appropriately as urban expansion and high density stable vegetation type (UE-HDSV). For cluster 7 and cluster 9, the normalized NTL lines had a growth trend (**Fig 7I** and **7K**), and the normalized NDVI lines exhibited a declining trend (**Fig 7J** and **7M**). Thus, the development trend type of cluster 7 and cluster 9 was defined appropriately as urban expansion and vegetation degeneration type (UE-VD).

**Fig 8:**
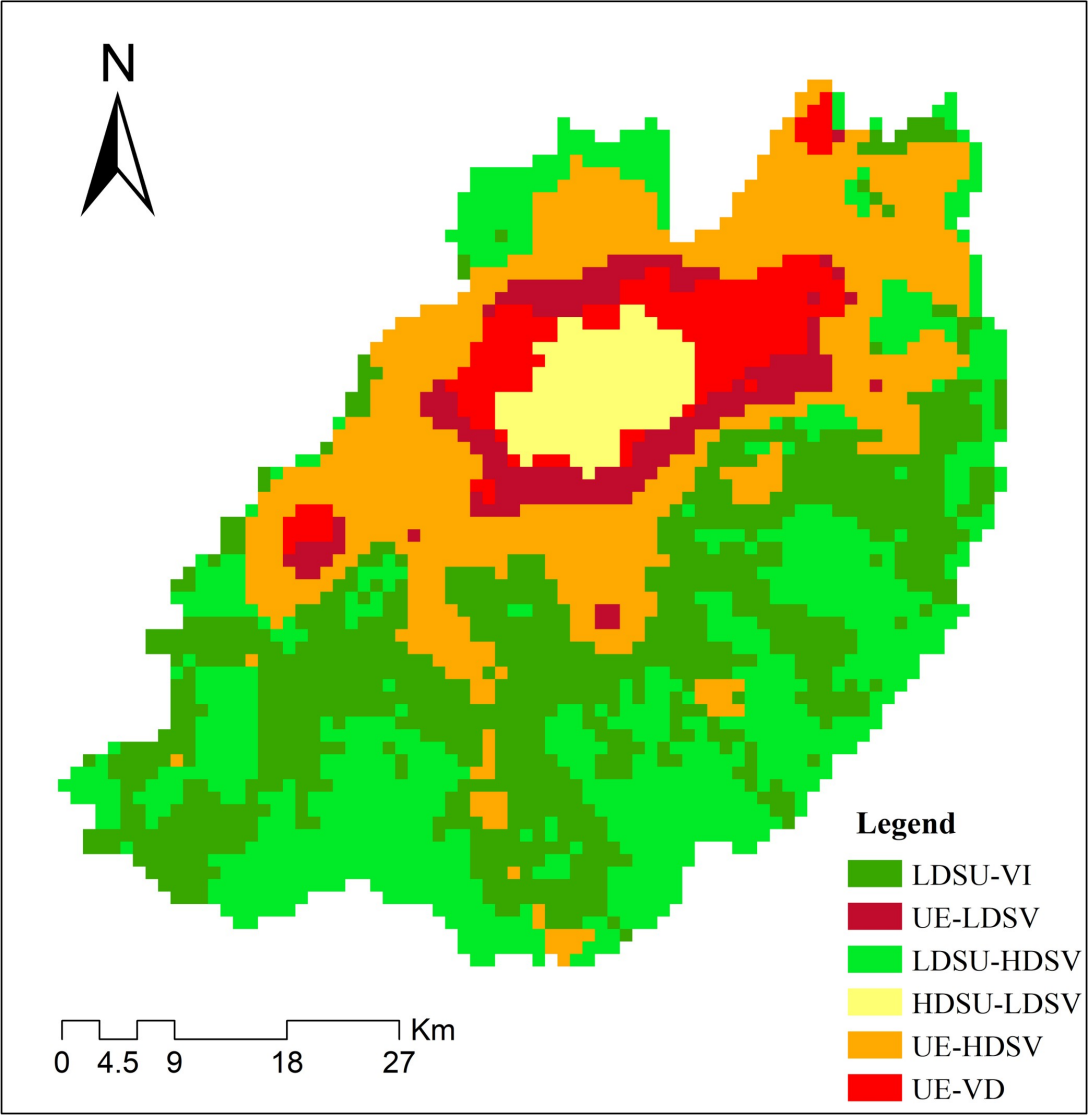
The urban development trend clustering results. LDSU-VI (type 1): Low density stable urban and vegetation increase type; UE-LDSV (type 2): Urban expansion and Low density stable vegetation type; LDSU-HDSV (type 3): Low density stable urban and High density stable vegetation type; HDSU-LDSV (type 4): High density stable urban and low density stable vegetation type; UE-HDSV (type 5): Urban expansion and high density stable vegetation type; UE-VD (type 6): Urban expansion and vegetation degeneration type.

As mentioned above, the twelve clusters were grouped into six development trend types based their linear model features. The spatial distribution pattern of the urban development types are shown in **Fig 8**. The area labeled HDSU-LDSV is the urban center. This high-density, stable urban area was already built-up, and there was little land available for urban expansion. The green areas labeled LDSU-VI and LDSU-HDSV were mainly at the edges of the study area. These regions are mainly occupied by forests and have been little influenced by urban development. Urban expansion mainly occurred in the regions labeled UE-LDSV, UE-HDSV, and UE-VD. However, in the UE-LDSV region, vegetation coverage remained low from 2000 to 2015. This is because this area is close to the center of Jinan and although it was not built up, the vegetation had already been disturbed by human activities. In the region UE-HDSV, vegetation coverage remained high from 2000 to 2015. This is because this region has a large area of farmland, and most urban land has changed from farmland, thus little forest have been urbanized. In the regions UE-VD, vegetation coverage degenerated rapidly during the study period. This is because these regions were close to prior city built-up areas and were antecedent areas of urban expansion. Urban construction activities were frequent, and the vegetation was seriously disturbed.

### Urban simulation

**Fig 9:**
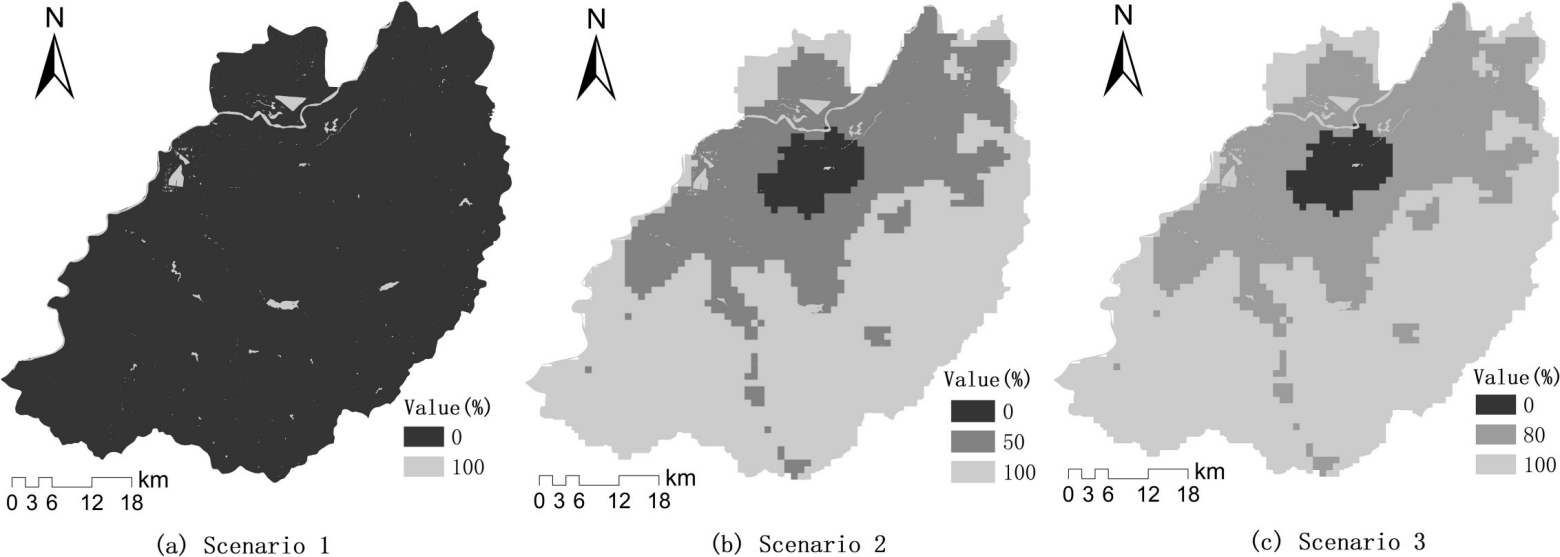
Exclusion layers for urban expansion scenarios.

Three scenarios were set by adjusting the exclusion layer values (Fig 9). The first scenario is called the historical growth scenario. In this scenario, only water bodies were completely excluded using a value of 100 in the exclusion layer with other areas set at zero. The second and third scenarios were built based on the results of the urban development trend analysis. The second scenario is called the gentle restriction scenario of urban expansion. In this scenario, in order to protect the ecological environment of the suburbs, in addition to water bodies, areas in the LDSU-VI and LDSU-HDSV classes were completely excluded (a value of 100). Urban expansion was allowed in regions UE-LDSV, UE-HDSV and UE-VD, their exclusion values were set at 50 to ensure the gentle restriction of urban growth. The third scenario is a strict urban expansion restriction scenario. In this scenario, the water bodies, LDSU-VI, and LDSU-HDSV were as in the second scenario. The regions UE-LDSV, UE-HDSV and UE-VD, were set to 80 in the exclusion layer to more strictly limit urban growth.

SLEUTH’s three calibration stages, coarse, fine, and final, were applied for each simulation scenario. As shown in Table 2, the optimal coefficients for dispersion, breed, spread, slope, and road gravity were 1, 18, 19, 90, and 75 in scenario 1; 1, 1, 39, 68, and 68 in scenario 2; and 1, 2, 78, 56, and 37 in scenario 3. The average Lee-Salle values of the three calibration processes were 0.707 in scenario 1, 0.709 in scenario 2, and 0.708 in scenario 3. According to previous research, calibration is acceptable when the Lee-Salle values are between 0.3 and 0.7 [24, 60]. The Lee-Salle values in this study were all higher than 0.7, which indicates a good calibration result.

**Table 2:** Calibration results of the three scenarios.

		Optimal coefficient combination		
		Scenario 1	Scenario 2	Scenario 3
**Model parameter**	Dispersion	1	1	1
	Breed	18	1	2
	Spread	19	39	78
	Slope	90	68	56
	Road gravity	75	68	37
**Average Lee-Sallee**		0.707	0.709	0.708

**Fig 10:**
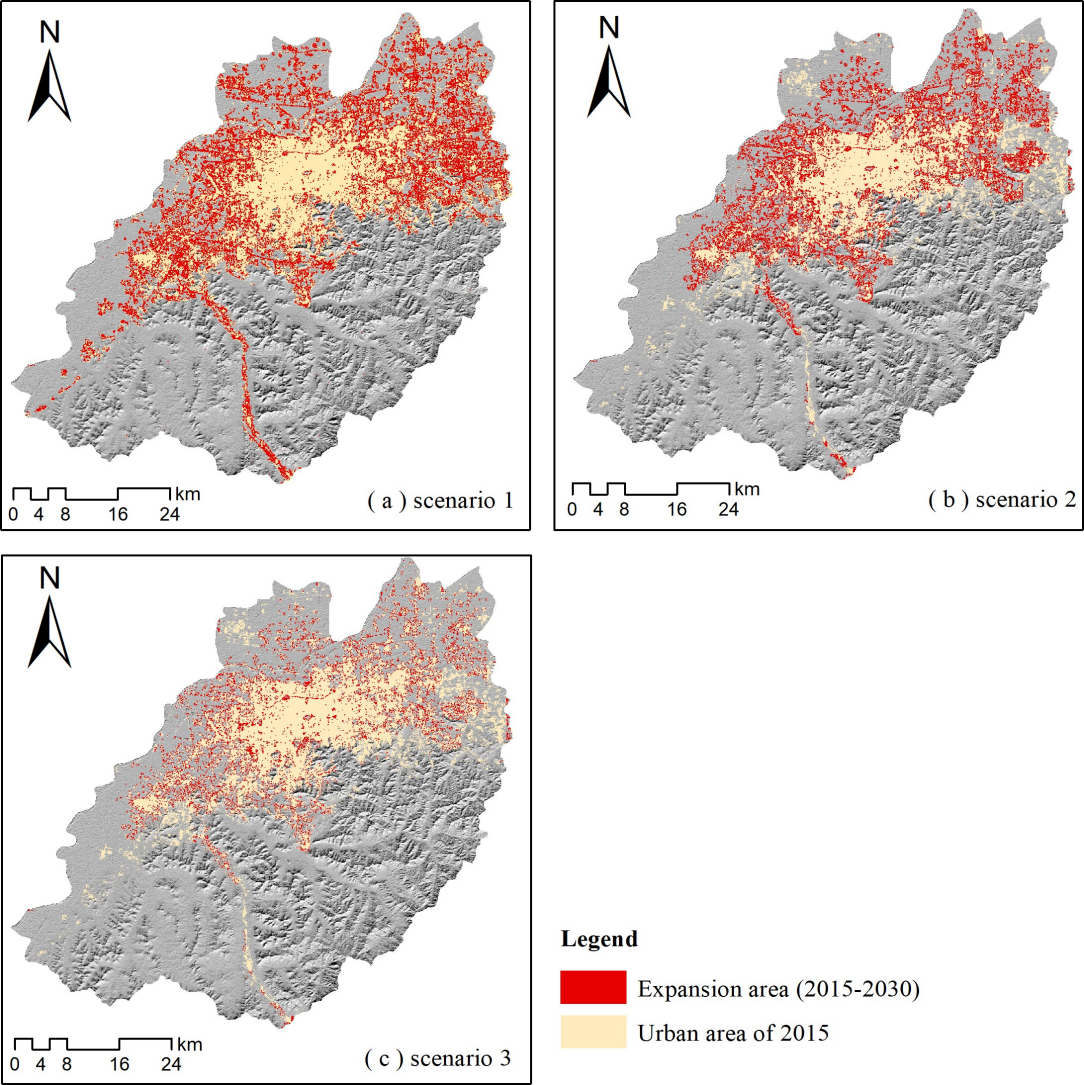
Urban land area predicted under three scenarios by 2030. (a) Scenario 1, historical growth; (b) Scenario 2, gentle restriction of urban expansion; (c) Scenario 3, strict restriction of urban expansion.

Using the 2015 urban area as the seed layer, this study predicted urban growth to 2030 under the three urban growth scenarios using the SLEUTH model (**Fig 10**). In the historical growth scenario (**Fig 10A**), the urban build-up land area would increase to 728.68 km^2^ in 2030, a gain of 104.5 km^2^ compared with 2015, with an average annual urban growth rate of 1.1% (Table 3). In this scenario, only water bodies were set as non-urbanizable in the exclusion layer. The restriction of urban expansion was the lowest across the three scenarios. Most urban expansion occurred adjacent to prior urban boundaries. Some newly added built-up lands occur along roads in the suburbs and connected scattered urban patches. The gentle restriction scenario of urban expansion (scenario 2) would add 58.5 km^2^ of build-up land by 2030, relative to 2015, with an average annual urban growth rate of 0.7%. In the strict restriction scenario, 22 km^2^ of built-up land would be added by 2030, with an average annual urban growth rate of 0.3%.

**Table 3:** Predicted urban expansion statistics, in 2030, under three scenarios.

Scenarios	Urban land(km^2^)	Urban growth land (km^2^)	Annual Urban growth rate (%)
**Scenario 1**	728.68	104.5	1.1
**Scenario 2**	682.62	58.5	0.7
**Scenario 3**	646.08	22	0.3

## Discussion

Time series NTL and NDVI data were used to identify change trends in urban land and nearby vegetation. A linear model of the change trend was built for each pixel based on the time series data. The parameters of the models, namely slope and intercept values, were used in k-means clustering to identify areas of homologous urban development trend type. Urban development spatial types were then used to establish three scenarios in an urban growth simulation, from 2015 to 2030.

In previous studies, time series NDVI and NTL data have been used for urban land mapping [36, 37] and urban land dynamic analysis [38–40]. Correlation analysis is commonly used to analyze the influence of urbanization on the eco-environment [38, 39]. This study used linear model fitting to analyze the trends of urbanization and ecology based on time series NDVI and NTL data. This can identify the rate of urbanization and ecological change, alongside the correlation information, through the parameters of the models. Next, to determine the spatial distributions of the urban development trend types, the parameters of the NTL and NDVI linear models were set as variables in a spatial clustering process. Finally, six urban development types were defined based on the clustering results and prior knowledge of the study area.

**Fig 11:**
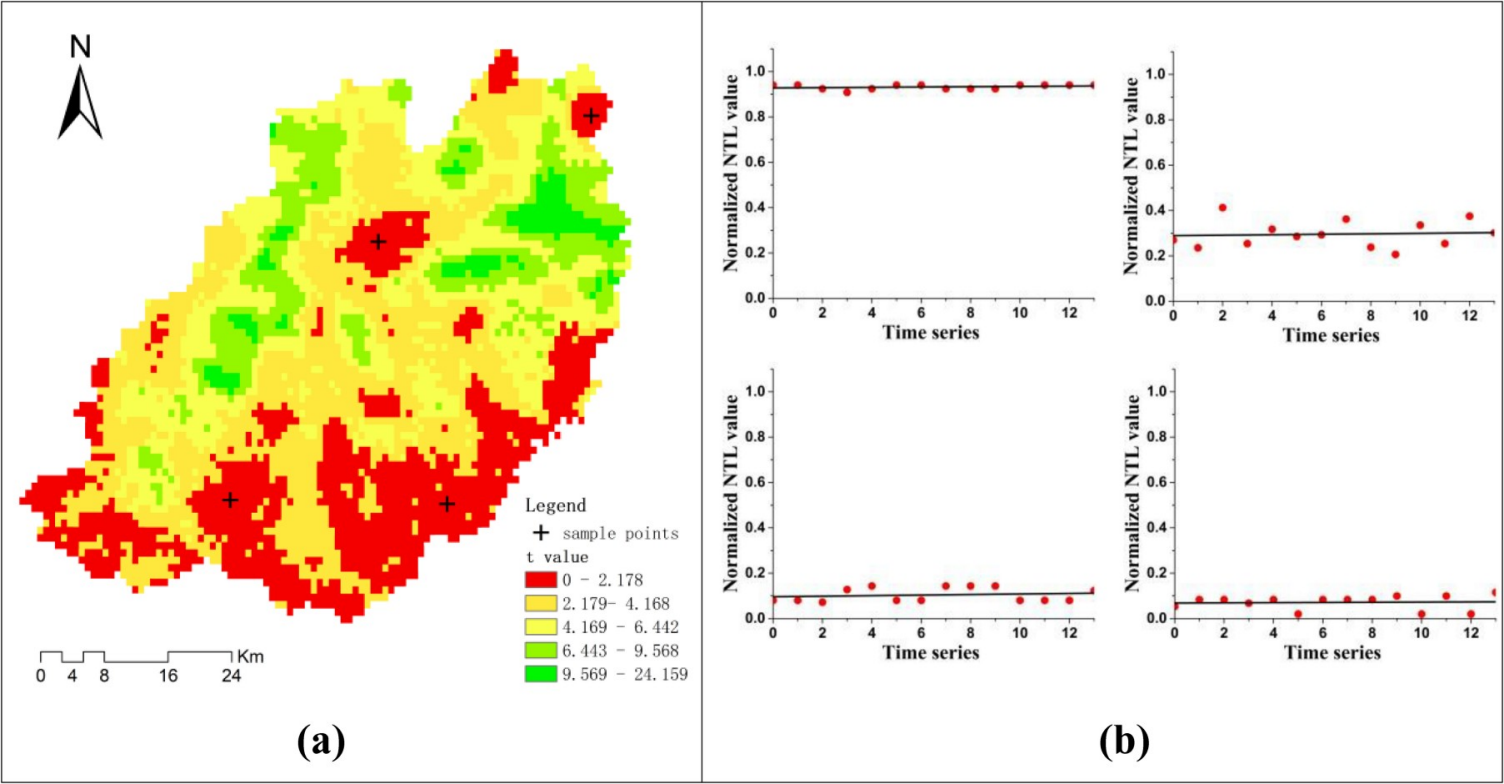
T-test values of time series normalized NTL linear fitting. (a) the spatial distribution map of t-test values. (b) examples of NTL linear fitting at sample points with low t-test values.

In linear fitting process, the goodness or the significance of linear fitting are usually extracted to prove the effectiveness of the linear models, and the related indicators contain root mean squared error (RMSE), Student’s T-test value, p-test value, and so on [49, 61]. In this study, we just selected the RMSE indicator to show the goodness of fitting and did not test the statistical significance of linear fitting. The reason is that the stable trends of NTL and NDVI is the important development pattern to dig out in this study, and they have a weak statistical significance when linear fitting due to the weak correlation between values and times. For example time series NTL data, at the significance level of 0.05, the significance threshold is 2.178. As shown in **Fig 11A**, the t-values of most regions are larger than 2.178. But in the red areas, the t-values are all less than 2.178. It means that the red areas have a weak statistical significance. And then, some sample points in these areas were selected randomly and showed the linear fitting results (**Fig 11B**). From these sample lines, we could find that the red areas are main NTL stable areas. It is inappropriate to validate the effectiveness of stable areas’ linear fitting results by statistical significance indicators.

We simulated urban growth up to 2030 using the SLEUTH model under three urban growth scenarios, which were built based on the results of the urban development trend analysis. The urban growth areas of the different scenarios were clearly affected by the degree of urban expansion restriction imposed by the exclusion layer. In the historical growth scenario (scenario 1), only the water bodies were excluded from urban expansion, and the urban growth speed was substantially higher than in the other two scenarios. Scenarios 2 and 3 were based on the concept of environmental protection. The regions of LDSU-VI and LDSU-HDSV were excluded in these scenarios by using the exclusion layer while growth in the main urban expansion areas, such as UE-LDSV, UE-HDSV, and UE-VD, was also appropriately restricted. Thus, the urban simulations of scenarios 2 and 3 directed urban growth with consideration of environmental protection and conservative urban development. The urban simulation process used in this study is therefore relatively simple. In future studies, it would be best to combine urban planning, land construction suitability, public will, and so on, into the urban development predictions [2].

Although this study successfully extracted the urban development trend types of Jinan and simulated urban growth in 2030, it had some limitations. First, only time series remote sensing data were used in the urban development analysis process, and it would be better to add statistical data about economic and social factors to identify urban development patterns in more detail. Second, in order to limit inconsistencies among different remote sensing data sources, this study only used the DMSP/OLS NTL data from 2000 to 2013, and it has stopped working and was substituted by data from the Visible Infrared Imaging Radiometer Suite (VIIRS) [62] after 2013. Third, this study simulated urban growth using the SLEUTH model. It is well known that some cellular automata-based urban simulation models are considered superior, such as the CA_MARKOV model [63], METRONAMICA model [64] and FLUS model [23]. Thus, it will be preferable to use these models in future urban simulation studies, especially if more socio-economic data is included. Finally, the urban growth scenarios were set based solely on the urban development analysis results, and it would be better to also include urban planning, land construction suitability [65], public will, and so on, in future urban development projections.

## Conclusions

Using time series DMSP/OLS NTL and NDVI data, we analyzed the urban development trends of Jinan from 2000 to 2015 and clustered these trends into six types. The first type was the low density stable urban and vegetation increase type (LDSU-VI). It was mainly distributed in southern Jinan, mostly mountains and forests. The second type was the urban expansion and low density stable vegetation type (UE-LDSV) mainly located in the suburban areas of Jinan. The third type was the low density stable urban and high density stable vegetation type (LDSU-HDSV) and is mainly in the south of Jinan. The fourth type was high density stable urban and low density stable vegetation type (HDSU-LDSV) mainly found in the center of Jinan. The fifth type was urban expansion and high-density stable vegetation (UE-HDSV) mainly in the suburbs of Jinan. The sixth type was urban expansion and vegetation degeneration (UE-VD) mainly in the near suburban areas of Jinan. Three urban growth simulation scenarios were built based on the urban development trend analysis and respecting the environmental protection and conservative urban development concepts. The urban simulations indicated a gentle urban growth trend from 2015 to 2030. The historical scenario had the highest urban growth rate. Scenarios 2 and 3 exhibited slower urban growth trends from 2015 to 2030 reflecting their different restriction levels. They show the prospects for urban growth from the perspective of environmental protection and conservative urban development.
